# An Insight into the Genesis of Hypohidrotic Ectodermal Dysplasia in a Case Report

**DOI:** 10.1155/2012/281074

**Published:** 2012-12-18

**Authors:** Kiran Kumar, Devi Charan Shetty, Mahima Dua, Amit Dua, Raghu Dhanapal

**Affiliations:** ^1^Department of Oral & Maxillofacial Pathology, I.T.S-CDSR, Muradnagar, Ghaziabad 201206, India; ^2^Department of Oral & Maxillofacial Pathology, Inderprastha Dental College, Sahibabad, Ghaziabad 201010, India; ^3^Department of Prosthodontics, Krishna Dental College, Mohannagar, Ghaziabad 201206, India

## Abstract

Hypohidrotic (anhidrotic) ectodermal dysplasia (HED) is a congenital syndrome characterized by sparse hair, oligodontia, and reduced sweating. It is estimated to affect at least one in 17000 people worldwide. We report a rare case of HED in a 14-year-old male child patient which extraorally manifested as alopecia, scanty eyebrow and eye lashes, frontal bossing, depressed nasal bridge, and full and everted lips. Intraoral examination revealed complete anodontia of the deciduous teeth and partial anodontia of the permanent teeth. It is usually inherited as an X-linked recessive trait caused by mutation in any of the three EDA pathway genes. X-linked and autosomal recessive forms are phenotypically similar; thus, identification of carriers of partial forms of the disorder in their families is the key to clarifying intrafamilial genetic transmission.

## 1. Introduction

Ectodermal dysplasia (ED) syndrome is a rare heterogeneous group of inherited disorders that share primary defects in the development of two or more tissues derived from ectoderm. These tissues primarily are the skin, hair, nails, eccrine glands, and teeth [1, 2]. Charles Darwin first documented the earliest accessible account of ectodermal dysplasia in English as early as 1840s. However, in the mid-19th century, it appeared that nobody understood the sex-linked inheritance and it was not until 1910 that this began to be understood [3]. To date, more than 150 distinctive syndromes have been described with all possible modes of inheritance. The most common syndromes within this group are hypohidrotic (anhidrotic) ED and hidrotic ED [1, 2]. 

Hypohidrotic ED (also known as Christ-Siemens-Touraine syndrome) is the most common phenotype [4]. Hypohidrotic (anhidrotic) ectodermal dysplasia (HED) is a congenital syndrome characterized by sparse hair, oligodontia, and reduced sweating [5]. Dental manifestations include conical or pegged teeth, hypodontia or complete anodontia, and delayed eruption of permanent teeth. 

It is usually inherited as an X-linked recessive trait [4]. It is caused by mutations in any of the three EDA pathway genes: ectodysplasin (EDA), EDAR, and EDARADD which encode a ligand, a receptor, and an intracellular signal mediator of a single linear pathway, respectively. In rare cases, HED is associated with immune deficiency caused by mutations in further downstream components of the EDA pathway that are necessary for the activation of the transcription factor NF-kappa b [5]. 

Morbidity and mortality are related to the absence or presence of eccrine and mucous glands [4]. Children with decreased sweating may have a mortality rate of up to 30% in infancy or early childhood because of intermittent hyperpyrexia [6]. No definite pharmacological treatment is available, and the management of affected patients depends on which structures are involved [4]. Patients with HED are advised light clothing, a cool-water spray bottle, air conditioning for environment, use of artificial tears, and application of petrolatum jelly for nasal mucosa protection [7]. Orthodontic treatment may be indicated for cosmetic reasons and to ensure adequate nutritional intake [7–9].

## 2. Case Report

A 14-year-old male child patient reported to the department of Oral & Maxillofacial Pathology with a chief complaint of absence of teeth since birth. His parents stated that he had difficulty in speaking and eating. He also had repeated episodes of unexplained hyperpyrexia. No contributory medical, family, or personal history was elucidated.

**Figure 1:**
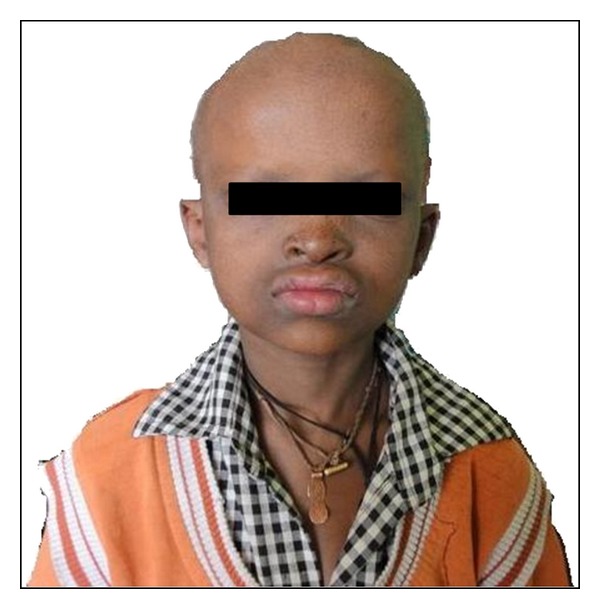
Photomicrograph showing fine, sparse scalp hair, scanty eyebrow and eye lashes, frontal bossing or depressed nasal bridge, and full and everted lips.

**Figure 2:**
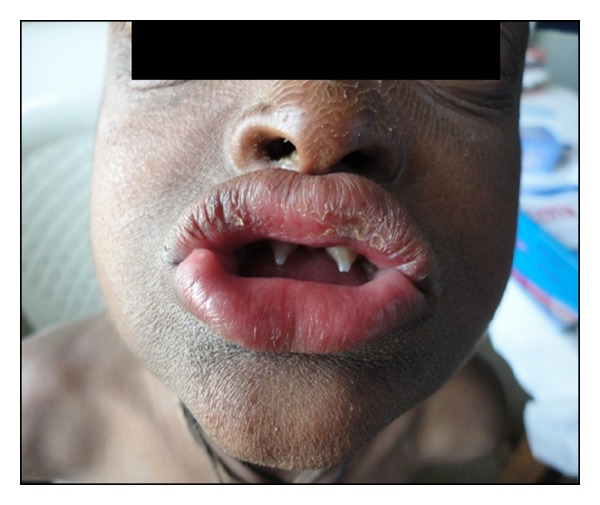
Photograph showing extraorally dry and eczematous rash on skin and intraorally conical shaped maxillary central incisors and a wide midline diastema and hypoplastic labial frenum.

On extraoral examination, patient presented with fine, sparse scalp hair, scanty eyebrow and eye lashes, frontal bossing, depressed nasal bridge, and full and everted lips (Figure 1), Dry eczematous (atopic) rash was seen on the face (Figure 2).

Intraoral examination revealed complete anodontia of the deciduous teeth and partial anodontia of the permanent. There was complete absence of mandibular teeth and partial anodontia in the maxillary arch. Teeth that were present were 11, 16, 21, and 26, widely spaced and pointed. Maxillary central incisors were conical shaped (Figure 2).

Soft tissue examination revealed a wide midline diastema and hypoplastic labial frenum (Figure 2).

Laboratory investigation shows that HED is diagnosed after infancy on the basis of physical features in most affected individuals. Carrier detection for X-linked HED was done by assessing the distribution and mosaic patterns of sweat pore function by the use of an iodine solution to assess sweat gland function or impression materials to assess number and distribution of sweat pores. The carriers for X-linked HED show this pattern, and 60% to 80% of carriers display some degree of hypodontia.

The mode of inheritance was further determined by family history. Diagnosis was confirmed by skin biopsy, which revealed thin and flattened epidermis, reduction in the number, and presence of rudimentary eccrine sweat glands.

Molecular genetic testing is clinically available for all three genes. Other newer methods are preimplantation genetic diagnosis (PGD) which is a means of detecting a specific genetic mutation within an embryo before it is transferred to the womb and forms a pregnancy. As the risk of having a child with a serious chromosomal abnormality increases significantly as you get older and this can be performed earlier, around the 11th week of pregnancy, CVS is preferred over amniocentesis

Treatment plan was emollient with topical steroid for his eczematous rash. Neither soap nor any perfumed bath additives, including baby bath products, were advised as these all have a drying effect and are irritating to the skin which may be sensitive. During the summer months individuals with fair skin should use a high factor sun block at all times, preferably one for sensitive skin. Cotton clothes next to the skin and cotton bedclothes were advisable.

## 3. Discussion

HED or anhidrotic ectodermal dysplasia is the most common syndrome among this large group of hereditary disorders. Hypohidrosis, hypotrichosis and hypodontia constitute the main symptoms of the syndrome [10]. HED affects at least one in 17000 people worldwide [10]. In the hypohidrotic form, the skin is soft, thin, and dry. The sebaceous glands are also defective or absent [11, 12]. Palms and soles are hyperkeratotic; pseudorhagades are present around the eyes. In the oral cavity, the most striking feature is oligodontia. The teeth that are present have abnormal crown form. Teeth in the anterior region of maxilla and mandible are conical in shape [12, 13]. There are a wide midline diastema and hypoplastic labial frenum in autosomal recessive condition where there is a total absence of permanent teeth with or without taurodontism of primary molars [14]. The characteristic facial features are frontal bossing, depressed nasal bridge, prominent supraorbital ridges, prominent and obliquely set ears, midface is depressed, the lower third of the face appears small due to lack of alveolar bone development, and lips are protuberant [11, 15]. A cephalometric study by Vierucci and coworkers [16] has shown that children with hypohidrotic type ectodermal dysplasia showed maxillary retrusion due to sagittally under developed maxilla, forward and upward displacement of the mandible, and collapsed lower anterior facial height [16]. The presentation of thin eyebrows, fine, stiff, and short scalp hair, scanty eyelashes and eyebrows, and partial anodontia observed in our case is in agreement with the existing literature.

 The gene responsible for X-linked HED is localized at Xq12-q13.1 and affects a transmembrane protein expressed by keratinocytes, hair follicles, and sweat glands, possibly having a key role in epithelial-mesenchymal signaling [17]. Two different ways of transmission of the disorder are known. Along with the more common X-linked recessive modality of transmission, a less frequent autosomal recessive one has been demonstrated [18]. In X-linked HED, the affected patients are most often hemizygous male subjects. Autosomal recessive HED is similar to the hemizygous form of X-linked HED from the clinical point of view except that males and females are equally affected. A gene responsible for autosomal HED has recently been mapped to chromosome 2q11-q13 [18].

The molecular pathogenesis of hypohidrotic ectodermal dysplasia (HED) is poorly understood. The gene responsible for X-linked HED, EDA, produces ectodysplasin-A, a protein that is important for normal development of ectodermal appendages including hair, teeth, and sweat glands. Evidence is accumulating that ectodysplasin-A is important in several pathways that involve ectodermal-mesodermal interactions during embryogenesis. Defects in the molecular structure of ectodysplasin-A may inhibit the action of enzymes necessary for normal development of the ectoderm and/or its interaction with the underlying mesoderm [19]. 

The protein encoded by *EDARADD* is similar to the death domain, MyD88, a cytoplasmic transducer of Toll/interleukin receptor signaling [20]. It also contains a Traf-binding consensus sequence. It is coexpressed with tumor necrosis factor receptor superfamily member EDAR in epithelial cells during the formation of hair follicles and teeth. It interacts with the death domain of EDAR and links the receptor to signaling pathways downstream [19].

Abnormal gene product: *EDARADD* mutations alter the charge of an amino acid in the resultant gene, rendering it incapable of performing its function [19].

Even though X-linked and autosomal recessive forms are phenotypically similar, identification of the way of transmission is mandatory to give reliable genetic counseling to the family and to address molecular studies. Complete examination of relatives of patients with HED and identification of carriers of partial forms of the disorder in their families are the key to clarifying intrafamilial genetic transmission [21]. No similar case of ectodermal dysplasia has been identified among the relatives, which suggests that the propositus was probably a fresh mutation or due to translocation of genes as was suggested in a few other literature [22].

DNA, being the prime source for diagnosis, should be stored (typically extracted from white blood cells) for possible future use which is known as DNA banking. It is likely that testing methodology and our understanding of genes, mutations, and diseases will improve in the future, and consideration should be given to banking DNA of affected individuals [19].

## 4. Conclusion

In developed countries diagnosis pertains to laboratory identification of genes [23] and mode of inheritance of mutant genes associated with recessively X chromosome or autosomal dominant or recessive. It is to be considered that an absence of a positive family history should not be a factor in causing any diagnostic dilemmas with respect to ectodermal dysplasia, a condition that shows multiple modes of inheritance.

## References

[1] Weech AA (1929). Hereditary ectodermal dysplasia (congenital ectodermal defect). *American Journal of Diseases of Children*.

[2] Solomon LM, Keuer EJ (1980). The ectodermal dysplasias. Problems of classification and some newer syndromes. *Archives of Dermatology*.

[3] Perry D

[4] Ul Bari A, Rahman SB (2007). Hypohidrotic ectodermal dysplasia: a case report and literature review. *Journal of Pakistan Association of Dermatologists*.

[5] Mikkola ML (2009). Molecular aspects of hypohidrotic ectodermal dysplasia. *American Journal of Medical Genetics A*.

[6] Berg D, Weingold DH, Abson KG, Olsen EA (1990). Sweating in ectodermal dysplasia syndromes. A review. *Archives of Dermatology*.

[7] Dhanrajani PJ, Jiffry AO (1998). Management of ectodermal dysplasia: a literature review. *Dental Update*.

[8] Till MJ, Marques AP (1992). Ectodermal dysplasia: treatment considerations and case reports. *Northwest Dentistry*.

[9] Sweeney IP, Ferguson JW, Heggie AA, Lucas JO (2005). Treatment outcomes for adolescent ectodermal dysplasia patients treated with dental implants. *International Journal of Paediatric Dentistry*.

[10] Varghese G, Sathyan P (2011). Hypohidrotic ectodermal dysplasia—a case study. *Oral & Maxillofacial Pathology Journal*.

[11] Suprabha BS (2002). Hereditary ectodermal dysplasia: a case report. *Journal of the Indian Society of Pedodontics and Preventive Dentistry*.

[12] Crawford PJM, Aldred MJ, Clarke A (1991). Clinical and radiographic dental findings in X linked hypohidrotic ectodermal dysplasia. *Journal of Medical Genetics*.

[13] Ramraje SN, Wasnik M, Momin YA (2009). Anhidrotic ectodermal dysplasia—a report of two cases. *Bombay Hospital Journal*.

[14] Rajendran R, Sivapathasundaram B (1983). *Shafer’s Textbook of Oral Pathology*.

[15] Shaw RM (1990). Prosthetic management of hypohydrotic ectodermal dysplasia with anodontia. Case report. *Australian Dental Journal*.

[16] Vierucci S, Baccetti T, Tollaro I (1994). Dental and craniofacial findings in hypohidrotic ectodermal dysplasia during the primary dentition phase. *The Journal of Clinical Pediatric Dentistry*.

[17] Kere J, Srivastava AK, Montonen O (1996). X-linked anhidrotic (hypohidrotic) ectodermal dysplasia is caused by mutation in a novel transmembrane protein. *Nature Genetics*.

[18] Munoz F, Lestringant G, Sybert V (1997). Definitive evidence for an autosomal recessive form of hypohidrotic ectodermal dysplasia clinically indistinguishable from the more common X-linked disorder. *American Journal of Human Genetics*.

[19] Wright JT, Grange DK, Richter MK (1993). *Hypohidrotic Ectodermal Dysplasia*.

[20] Headon DJ, Emmal SA, Ferguson BM (2001). Gene defect in ectodermal dysplasia implicates a death domain adapter in development. *Nature*.

[21] Cambiaghi S, Restano L, Pääkkönen K, Caputo R, Kere J (2000). Clinical findings in mosaic carriers of hypohidrotic ectodermal dysplasia. *Archives of Dermatology*.

[22] Sharma J, Mamatha GP (2008). Hereditary ectodermal dysplasia: diagnostic dilemmas. *Revista de Clínica e Pesquisa Odontológica*.

[23] Kumar A, Eby MT, Sinha S, Jasmin A, Chaudhary PM (2001). The ectodermal dysplasia receptor activates the nuclear factor-*κ*B, JNK, and cell death pathways and binds to ectodysplasin A. *Journal of Biological Chemistry*.

